# Hydrothermal liquefaction of sewage sludge: use of HCOOH and KOH to improve the slurry pumpability in a continuously operated plant

**DOI:** 10.1016/j.heliyon.2024.e26287

**Published:** 2024-02-10

**Authors:** C. Prestigiacomo, Y. Fan, U. Hornung, N. Dahmen, O. Scialdone, A. Galia

**Affiliations:** a Dipartimento di Ingegneria, Università degli Studi di Palermo, Palermo, Italy; b Institut für Katalyseforschung und -technologie, Karlsruher Institut für Technologie (IKFT), Karlsruhe, Germany; c Nanyang Institute of Technology, School of Civil Engineering, Nanyang, 473004, PR China

**Keywords:** Hydrothermal liquefaction, Sewage sludge, Continuous plant, Wastes valorization, Biofeedstocks

## Abstract

We studied the hydrothermal liquefaction (HTL) of digested sewage sludge (DSS) as model of waste biomass in batch and continuous reactors. HCOOH and KOH were used to improve the slurry pumpability.

HTL experiments were conducted at the same kinetic severity factor in a batch reactor of 25 mL of volume and in a continuously operated tubular reactor with 350 mL of volume. The observed outcomes suggested that it was not possible to achieve the pumpability of native DSS when a high concentrated stream of suspended solid particles has been fed to the HTL continuous plant. Using acidic or basic homogeneous additives, as potassium hydroxide or formic acid, it was possible to enhance the pumpability of a concentrated slurry of DSS in the continuous plant achieving yields of heavy oil (fraction of biocrude) similar to those obtained in the batch reactor and with higher H/C ratios. Hence, we found that HCOOH and KOH are promising additives for the practical implementation of a continuous HTL process.

## Highlights

• Challenge of HTL of DSS from batch to continuously operated reactors.
• KOH and HCOOH enhance the pumpability of DSS in the continuous plant.
• Similar HO and SR yields in both types of reactors at the same KSF.

## 1 Introduction

In recent years, in which the ecological and energetic transitions represent the main challenges that drive the technological development of the industries, the hydrothermal liquefaction (HTL) of biomass is considered a potential advantageous technology for the production of biocrude (BC) as green energy carrier [1,2]. HTL takes place within a temperature range of 300–400 °C and a pressure range of 10–40 MPa [[3], [4], [5], [6]]. It is usually conducted using wet feedstocks because the water of the matrix constitutes a solvent and a co-reagent beside being a catalyst of hydrolytic reactions. The process produces four different phases, i.e., a gas phase that is enriched in carbon oxides, a residual solid phase, that is due to the non-converted feedstock and carbonisation products, and two immiscible liquid phases: one represented by the biocrude and the other one by the aqueous phase in which some of the light organic molecules can be solubilized. The BC produced from HTL is regarded as one of the potential alternatives to substitute the current reliance on fossil fuels and achieve the greenhouse gas reduction target. A recent conceptual techno-economic analysis performed by Li et al. [7] showed that the minimum selling price of BC obtained by the HTL of sewage sludge and manure is 1.70 $/equivalent gallon. Moreover, HTL process is energy intensive [[8], [9], [10]]. One possible technological improvement can be represented by the use of solar heat to drive the process. In the framework of an analysis of the HTL of microalgae assisted by solar heat, it was observed that the high cost of production of microalgae, one of the well-studied feedstock, hurdles the economic sustainability of the process [11]. The same authors demonstrated that the minimum fuel selling price of the BC can be reduced by 38 % if the solar HTL is performed with a zero-cost (waste) raw material instead of microalgae. Quite interestingly, several studies demonstrated that sewage sludge (SS), a wet waste biomass that can be readily available at no cost, could be utilized as viable alternative to microalgae with comparable biocrude (BC) productivity in batch experiments [[12], [13], [14], [15]].

The results of the theoretical assessment previously done with microalgae and the need to find out proper conditions for the practical feasibility of the liquefaction process inspired us to investigate the transfer of HTL of digested sewage sludge (DSS) from batch to continuously operated reactors mimicking industrial plant operations. The definition of a kinetic severity factor (KSF) was widely used to investigate the combined effect of reaction temperature and time during HTL processes [[16], [17], [18], [19], [20], [21]]. In the framework of thermochemical conversion of biomass the KSF was defined for the first time by Overend et al. [21] which demonstrated that reaction time and temperature can be effectively combined to yield comparable experimental outcomes. This assumption is based to the fact that the rate of reaction is directly proportional to the concentration of the reactants, indicating a first-order dependence and the apparent activation energy can be described by Arrhenius equation.

The KSF value can be obtained in integral form as in equation (1):

(1) $$KSF=\mathrm{Log}\;\left(R^{0}\right)=\mathrm{Log}\;\left(\int_{t0}^{tf}e^{\left(\frac{T_{r}-100}{14.75}\right)}dt\right)$$

In this framework experimental trials were conducted in batch and in continuously operated reactors at the same KSF to investigate the effect of a transfer of the process from batch reactors to a continuous plant.

It must be underlined that one of the main hurdles of the HTL in continuous reactors is the long term pumpability of a slurry with high concentration of suspended solids. The solution of this problem is made more complex by the variability of the composition of the DSS during the year that brings to significant variation of the concentration of suspended solid particles that when becomes too high strongly increases the possibility of plugging of the lines [7,22]. To this purpose, in the work herein xanthan gum, HCOOH and KOH were investigated as homogeneous additives to enhance the pumpability. Xanthan gum was used with microalgae slurry to mitigate the precipitation of the particles inside the pump [23]. HCOOH and KOH are widely studied as homogeneous additives in HTL showing effect on improving BC yield and quality and decreasing solid residue yield [16,17].

## 2 Materials and methods

### 2.1 Materials

Digested sewage sludge (DSS) utilized in the HTL experiments were obtained from the wastewater treatment plant (WWTP) located in Karlsruhe (Germany). The DSS had an initial moisture of 75 %w/w, with the properties listed in Table 1. The raw matrices were dewatered inside an oven at a temperature of 105 °C for 72 h, then they were milled with a pulverisette 14, Fritsch instrument and filtered through a sieve (60 mesh). The ash content was quantified using gravimetric measurements following a calcination process at a temperature of 550 °C for a duration of 6 h. The organic content was then calculated as the difference between the total mass and the ash content. In the work herein we adopted as additives potassium hydroxide of analytical grade purity provided by Acros Organics and formic acid (Fluka, 98–100 % w/w). The implementation of the continuously operated plant involved the utilization of Xanthan gum (Sigma Aldrich). HTL trials were conducted adopting deionized water as solvent. The experimental trials were conducted using a slurry with a concentration from 3 to 10 %w/w of dry suspended particles of DSS as feedstock. Furthermore the homogeneous additive was loaded with a concentration of 10 %w/w (dry DSS bases). The solvents used for the recovery of the BC were acetone (≥99.8%, HiPerSolv CHROMANORM® for HPLC) and cyclohexane (99.5 % HiPerSolv CHROMANORM® analytical grade). Gas chromatographic analyses of the collected liquid phases were conducted preparing samples diluted in tetrahydrofuran (THF) (Sigma-Aldrich, analytical grade ≥99.0 %) adopted as solvent. Internal standard methodology was used to quantify detected hydrocarbons and methyl heptadecanoate (Sigma Aldrich, analytical standard) was used as standard. The hydrocarbons peaks (C10–C18) were identified using analytical standards provided by Alpha Aesar.

**Table 1:** List of properties of dry DSS used in HTL runs.

Proximate analysis (% w/w db^a^)	DSS
Organic content^b^	60
Elemental analysis (% w/w db)	
C	29.2
H	4.8
N	4.3
S	1
HHV (MJ/kg)^c^	13
Al	1.12
Ca	3.66
Fe	7.32
K	<0.9
Mg	0.38
Na	<0.46
P	3.59
Ti	0.12
Zn	0.08
Si	3.39

a db: dry basis.

b the ashes % are determined by difference.

c HHV: higher heating value determined by Dulong's formula.

### 2.2 Experimental methods

HTL experiments were conducted at the reaction temperature of about 350 °C adopted in the conceptual analysis of HTL of microalgae driven by a concentrating solar power plant in Giaconia et al. [11]. The KSF was used as parameter to make comparable experiments in batch and in the continuously operated reactors and to calculate this factor, a numerical integration of eq. (1) has been performed. HTL was conducted in both types of reactors at the same KSF of 8.3.

**Fig. 1:**
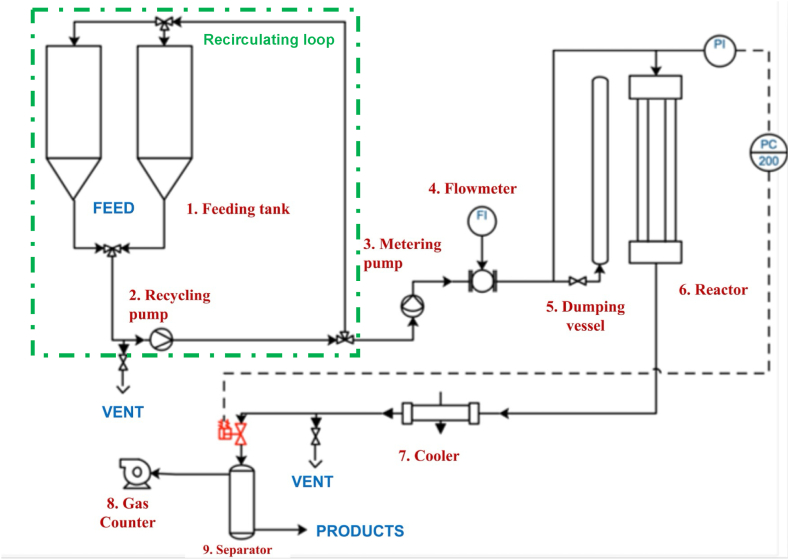
Process Flow Diagram of the continuous system.

10 g of slurry were loaded in the AISI 316Ti high-pressure batch reactor (25 mL of volume). The batch reactor was heated using a sand bath with a heating rate of the reaction mixture of about 30 °C/min. Given the fast heating rate, values of KSF decreased by only 0.3% when the heating ramp of the reactor was neglected. The continuously operated HTL plant (showed in Fig. 1) was implemented at the Karlsruhe Institut of Technology as described by Fan et al. [23]. It was equipped with a tubular reactor (350 mL of volume) that worked at 20 MPa and 350 °C and was build up by two feeding tanks (1), a recirculation piston pump that was used to recirculate the slurry and prevent sedimentation issues (2), a high pressure diaphragm pump (NOVADOS BRAUN LUEBBE NP31) (3), a Coriolis flowmeter (PROMASS 80F)(4), a pressurized vessel to minimize the pressure deviation inside the plant during the experimental runs (5), the tubular reactor (6), a cooler that allow to quench the stream (7), a normally open electropneumatic control valve of 3/8" (provided by SITEC) (8), a vessel to collect the condensable products and a gas counter (Rigamo) (9).

The continuously operated plant was controlled by a WinCC software that permitted the configuration of the operating parameters of the plant. The combined flow rate of the compressed water and feedstock mixture was set to 2 kg/h, resulting in a nominal residence time of 10 min. The system was previously tested pumping deionized water compressed at 25 MPa achieving different temperatures from 20 to 350 °C. The pre-run of an HTL experiment was performed feeding deionized water, to heat the reactor and reach a stable and uniform temperature of the stream in the tubular reactor. The reactor temperature was increased stepwise by 50 °C increments, with an heating rate between 1.5 and 3 °C/min, up to the desired reaction temperature. After this heating procedure, we had a total test duration of around 1–1.5 h. Prior to start each HTL run, we performed an initial period of conditioning lasting at least for three average residence time which was reported to be long enough to approach steady state conditions as reported by Wądrzyk et al. [24]. Sample collection was started 30 min after the beginning of the test, switching the feed from water to DSS suspension in the steady state condition.

Xanthan gum, KOH and HCOOH, added to the feed at 10 %w/w (dried DSS basis), were investigated as homogeneous additives to enhance the pumpability of the stream and stabilize the flow-rate. The ongoing operability of the HTL plant was tested for a maximum time of 30 min.

After each experiment in the continuously operated plant, the condensable produced phases (liquids and solid) that are firstly collected in the tank (9) in Fig. 1, were thereafter transferred in a glass separatory funnel.

The adopted separation procedures are reported in previous works [16,17,25,26]. Briefly, after the collection in the separatory glass funnel, almost 50 mL of cyclohexane were added to isolate the upper drops of oily phase from aqueous phase. The cyclohexane rich-phase was recovered with Pasteur pipettes and collected in a glass rounded bottom flask. The remaining phase rich in water was then recovered using a glass pipette and two samples of it were centrifuged at 3200 rpm for 10 min two times to recover and quantify the suspended particles. Acetone was then added to the separatory funnel to fluidize the solid particles and recover the oily phase. The obtained suspension was then filtered under vacuum atmosphere to extract the oily phase containing the BC.

Cyclohexane and acetone solutions were mixed and a controlled evaporation was performed using the apparatus described elsewhere [26]. In the bottom of the rounded bottom tank the heavy oil (HO) fraction was recovered, in the cooling section a condensed solution containing hydrocarbon (HC) fractions was collected.

### 2.3 Analytical methods

The proximate analyses of DSS were determined by gravimetric methodology determination. CHNS elemental analyses were conducted using an Elementar vario MICRO cube. O fraction (% w/w) was calculated by difference (as in eq. (2)).

(2) O=100−C−H−N−S−ash

All HTL experimental runs were conducted twice to determine the reproducibility and reported yields are mean values. Standard deviations of the product yields with the batch reactor are: 3 percentage points for heavy oil (HO); 4 for solid residue (SR); 0.5 for water soluble products (WSP) and 5 for Gas. The terms biocrude (BC) yield was used to indicate the sum of the HO and HC fraction yields. The yields of HO, HC, WSP, SR and Gas ($Y_{product}(\%$)) are calculated following to eq. (3) in the dry ash free formula (daf):

(3) $$Y_{product}\left(\%\;w/w\right)=M_{p\left(daf\right)}/M_{b\left(daf\right)}\times 100$$

Where $M_{p\left(daf\right)}$ is the daf mass of product and $M_{b\left(daf\right)}$ is the daf mass of matrix charged into the reactor at the beginning of the experiment .The daf mass of SR and WSP was obtained by drying two samples at 60 °C over the night and calcinating the dried samples at 550 °C for 6 h.

When formic acid was used as additive its mass was included in the mass of the initial organic matrix since HCOOH itself reacts inside the reactor at the reaction temperature [27,28].

The solutions recovered after solvent evaporation inside the trap were collected and analyzed in a gas chromatograph Perkin Elmer Autosystem XL. A capillary column ZB-FFAP (30 m ∗ 0.25 mm ∗ 0.25 mm) Phenomenex was used to characterize the hydrocarbon fraction. The oven program consisted of an initial temperature of 40 °C maintained for 3 min, followed by a heating rate of 8 °C/min until 244 °C, which was maintained for 0 min. The temperature of the injector and the FID detector was set at 250 °C. The split ratio employed in the experiment was 15:1, the helium flow-rate whitin the column was maintained 2.4 mL/min. Additionally, the injection volume of the sample was 0.2 μL. GC analytical standards were adopted to identify the peaks in the chromatograms and to build the calibration for each compound.

The higher heating values (HHV) of the dry matrices and of the HO were obtained by the Dulong formula reported in eq. (4):

(4) HHV(MJ/kg)=0.338C+1.44(H–O/8)+0.094S

HO productivity was calculated by doing the ratio between the collected product mass and the run time (30 min) of the continuously operated reactor.

## 3 Results and discussions

In the work herein, experimental trials were first performed in batch reactors. Then, the continuously operated reactor was tested to conduct the HTL of DSS maintaining the same KSF of that used in batch runs to make comparable the behavior of the process considering the product yields [6]. The first experimental trials were carried out with the aim to investigate the pure thermal HTL of DSS (without any additive) in batch and continuous reactors loading a slurry composed by 10 %w/w of dry DSS in water. The main data are reported in Table 2 (Experiments 1 and 4).

**Table 2:** HTL experiments of DSS at 350 °C, 10 min conducted in batch and continuously operated reactors in the presence of KOH, HCOOH and Xanthan gum as homogeneous additives and different DSS concentration in water.

Exp.	Reactor	Homogeneous additive	DSS concentration (% w/w)	Product yields (daf %w/w)					Elemental analysis of HO			HO productivity (g/h)
				HO	SR	WSP	HC	Gas	H/C	O/C	HHV (MJ/kg)	
1	B^a^	_	10	25	17	11	4	7	1.51	0.12	36	_
2	B^a^	KOH	10	23	8	16	8	10	1.53	0.12	37	_
3	B^a^	HCOOH	10	25	6	7	4	10	1.53	0.13	36	_
4	CO^b^	_	10	ND^c^	ND^c^	ND^c^	ND^c^	_	ND^c^	ND^c^	ND^c^	ND^c^
5	CO^b^	_	3	13	8	10	ND	_	_	_	_	5
6	CO^b^	Xanthan gum	10	ND^c^	ND^c^	ND^c^	ND^c^	_	ND^c^	ND^c^	ND^c^	ND^c^
7	CO^b^	KOH	10	23	4	11	5	_	1.64	0.14	36	33
8	CO^b^	HCOOH	10	24	6	10	<1	_	1.65	0.09	40	35

a B: batch reactor.

b CO: continuously operated reactor.

c The pumping of the feed was not possible.

When the experiment was performed in the continuously operated reactor the check valves of the metering pump (Fig. 1, element 3) underwent immediate blockage hence impeding the generation of pressure in the system.

From this standpoint, many efforts were done in order to find the optimal operational parameters to feed a high pressurized DSS suspension to the tubular reactor. A first hypothesis was that the blockage could be due to the localized accumulation of solid particles entrained in the DSS/water slurry. The flow rate in the recirculating loop was chosen to guarantee effective mixing in the tank and to limit the settling of particles in the pipelines leading to the metering pump. The presence of a fibrous fraction in the suspension poses significant challenges, as confirmed by the study of Shah et al. [29]. In fact the fibrous material can become trapped in the non-return valves and create a filter-like structure that accumulates additional suspended solid material over time. To overcome this drawback, it would be necessary to enhance the turbulence by increasing the flow rate. In the circulation line upstream of the metering pump, this can be easily achieved. In the pressurized reactor line this strategy is limited by the inverse correlation between flow rate and residence time: higher flow rate would significantly decrease the reaction time. The accumulation of solid material must be affected by the concentration of DSS. A dilution of the feed was implemented to validate this hypothesis. When the DSS concentration in the feed decreased from 10 % to 3 % the experiments were conducted successfully, without any blockage (experiment 5 in Table 2). However, the obtained biocrude productivity (in terms of HO) was too low (5 g/h) and the HO was difficult to separate from the produced phases so that this solution is not economically sustainable for a continuous HTL process that requires as feed a high concentration slurry.

To approach this condition we tested the possibility of using a surfactant to avoid the localized sedimentation of the concentrated slurry and Xanthan gum was used at a loading of 0.1, 0.5 and 1 %w/w. This methodology was previously used by Barreiro et al. [23] to prevent the sedimentation of microalgae slurry during the pumping in a continuously stirred reactor. However, in the case of DSS, this strategy did not work as in all tests when the concentration of the slurry was 10 % w/w, the localized sedimentation in the non-return valves downstream of the metering pump occurred again, hindering the pressurization of the entire plant (experiment 6 in Table 2). After this result we tested a novel strategy based on the addition of KOH and HCOOH as basic and acidic additive in the tank of the feed. This route was selected because these additives decrease the solid residues yield in batch reactors as observed in previous works [16,17]. Furthermore, it was also reported that the same homogeneous additives can be effective in the improvement of the biocrude quality acting as hydrogen donors [16,17].

The effects of the addition to DSS of KOH and HCOOH was evaluated in batch experimental trials (Table 2), that confirmed the reduction of solid yield, which decreased from 17 to 8 and 6% with KOH and HCOOH respectively.

On the basis of these outcomes, HTL of DSS in the continuously operated plant was then conducted in the presence of KOH or HCOOH mixed with the initial feed. In these experiments we observed a strong improvement of the pumping time (experiments 7 and 8 in Table 2). In the literature it is reported that basic or acidic solution may interact following different pathways with organic matter of the feedstock. Dido et al. [30] suggested that the addition of formic acid suppresses the precipitation of lignin by forming formate esters with its hydroxyl groups, thus preventing further condensation [31] at high temperature. Alternatively, it is possible that in acidic conditions, formic acid in its protonated form may generate a formyl cation [32], which could react with the aromatic ring of dissolved lignin, reducing its reactivity to combine with another soluble lignin fragment. This reduced reactivity can be due to the electron-withdrawing properties of the formyl group. These studies and our results coherently support the hypothesis that KOH and HCOOH do not act as a surfactant, but they chemically interact with the organic matter of the feedstock decreasing the amount of solid matter thus assisting its pumping. The HO and SR yields obtained in the tubular reactor were similar to those achieved in batch reactors (experiments 2,3,7 and 8 in Table 2). Moreover in the case of tubular reactor a smaller hydrocarbon fraction was detected probably because with the continuous plant configuration they are lost in the separator (Element 8 in Fig. 1). The higher H/C molar ratio of HO obtained from the continuous reactor with respect to that recovered from the batch system suggests that the different fluid dynamic regime can assist the hydrogenation activity of the homogeneous additives [16,17].

## 4 Conclusion

The main drawback to ongoing running of HTL of DSS is constituted by the inadequate stability of the flow rate and the challenges in control of the valve wear hindering the operability of the system.

In particular we observed that the transfer of HTL of DSS from batch to continuously operated reactors can be affected by the amount of suspended solid particles in the stream. The reduction of the initial feed concentration from 10% to 3% resulted in a more stable flow-rate. However, this adjustment led to a decrease in HO productivity to 5 g/h, incompatible with applicative valorization of the process. Quite interestingly it was observed that basic or acid compounds (as potassium hydroxide or formic acid), added in the pretreatment step, allowed to obtain similar HO and SR yields in both types of reactors at the same kinetic severity factor and they seem promising additives to feed a slurry at 10 %w/w of concentration for longer period of time.

## Data availability statement

Data included in article/supplementary material/referenced in article.

## CRediT authorship contribution statement

**C. Prestigiacomo:** Writing – review & editing, Writing – original draft, Investigation, Data curation, Conceptualization. **Y. Fan:** Writing – review & editing, Methodology, Investigation. **U. Hornung:** Writing – review & editing, Visualization, Supervision. **N. Dahmen:** Writing – review & editing, Visualization. **O. Scialdone:** Visualization. **A. Galia:** Writing – review & editing, Supervision, Funding acquisition.

## Declaration of competing interest

The authors declare that they have no known competing financial interests or personal relationships that could have appeared to influence the work reported in this paper.
